# Supplementary material for: Effects of far infrared therapy in hemodialysis arterio-venous fistula maturation: A meta-analysis
Source: PLoS One. 2024 Aug 28;19(8):e0307586. doi: 10.1371/journal.pone.0307586 (PMC11356441; doi:10.1371/journal.pone.0307586)
Supplement: S1 File — (DOCX) [file pone.0307586.s002.docx]

Fig 3. Forest plot of comparison: 1 Far infra-Red phototherapy for AVF malformation, outcome: 1.5 Physiologic maturation of AVF at 3mo.

| Study or Subgroup | FIR | | Control | |
| --- | --- | --- | --- | --- |
|  | Events | Total | Events | Total |
| Lin 2013 | 54 | 60 | 47 | 62 |
| Chen 2022 | 44 | 50 | 35 | 51 |

Fig 4. Forest plot of comparison: 1 Far infra-Red phototherapy for AVF malformation, outcome: 1.6 Clinical maturation of AVF within 12mo.

| Study or Subgroup | FIR | | Control | |
| --- | --- | --- | --- | --- |
|  | Events | Total | Events | Total |
| Lin 2013 | 49 | 60 | 37 | 62 |
| Chen 2022 | 42 | 50 | 32 | 51 |

Fig 5. Forest plot of comparison: 1 Far infra-Red phototherapy for AVF malformation, outcome: 1.4 Unassisted patency of AVF at 12 mo.

| Study or Subgroup | FIR | | Control | |
| --- | --- | --- | --- | --- |
|  | Events | Total | Events | Total |
| Lin 2013 | 52 | 60 | 43 | 62 |
| Chen 2022 | 42 | 50 | 33 | 51 |

Fig 6. Forest plot of comparison: 1 Far infra-Red phototherapy for AVF malformation, outcome: 1.7 Assessment of Qa0 (mL/min)

| Study or Subgroup | FIR | | Control | |
| --- | --- | --- | --- | --- |
|  | Events | Total | Events | Total |
| Lin 2013 | 256.5 | 81.0 | 60 | 259.4 |
| Chen 2022 | 302.2 | 110.1 | 50 | 301.0 |

Fig 7. Forest plot of comparison: 1 Far infra-Red phototherapy for AVF malformation, outcome: 1.8 Assessment of Qa1 (mL/min)

| Study or Subgroup | FIR | | Control | |
| --- | --- | --- | --- | --- |
|  | Events | Total | Events | Total |
| Lin 2007 | 975.2 | 421.9 | 63 | 992.8 |
| Lin 2013 | 700.7 | 287.3 | 60 | 582.2 |
| Murugesh Anand 2020 | 714.1 | 221.5 | 51 | 581.3 |
| Chen 2022 | 761.8 | 276.9 | 50 | 596.5 |

Fig 8. Forest plot of comparison: 1 Far infra-Red phototherapy for AVF malformation, outcome: 1.9 Assessment of Qa2 (mL/min)

| Study or Subgroup | FIR | | Control | |
| --- | --- | --- | --- | --- |
|  | Events | Total | Events | Total |
| Lin 2007 | 948.6 | 432.7 | 63 | 975.9 |
| Lin 2013 | 847.8 | 307.6 | 60 | 678.8 |

Fig 9. Forest plot of comparison: 1 Far infra-Red phototherapy for AVF malformation, outcome: 1.10 Assessment of Qa3 (mL/min)

| Study or Subgroup | FIR | | Control | |
| --- | --- | --- | --- | --- |
|  | Events | Total | Events | Total |
| Lin 2007 | 1011.6 | 447.0 | 63 | 941.1 |
| Lin 2013 | 1001.1 | 380.9 | 60 | 802.0 |
| Murugesh Anand 2020 | 850.4 | 316.1 | 51 | 649.8 |
| Chen 2022 | 982.2 | 299.4 | 50 | 787.5 |

Fig 10. Forest plot of comparison: 1 Far infra-Red phototherapy for AVF malformation, outcome: 1.11 Assessment of Qa4 (mL/min)

| Study or Subgroup | FIR | | Control | |
| --- | --- | --- | --- | --- |
|  | Events | Total | Events | Total |
| Lin 2007 | 1130.8 | 344.3 | 60 | 915.6 |
| Chen 2022 | 1089.4 | 376.2 | 50 | 84.2 |

Fig 11. Forest plot of comparison: 1 Far infra-Red phototherapy for AVF malfunction, outcome: 1.1 AVF malfunction.

| Study or Subgroup | FIR | | Control | |
| --- | --- | --- | --- | --- |
|  | Events | Total | Events | Total |
| Lin 2007 | 9 | 72 | 22 | 73 |
| Lin 2013 | 7 | 60 | 18 | 62 |
| Chen 2022 | 8 | 50 | 18 | 51 |

Fig 12. Forest plot of comparison: 1 Far infra-Red phototherapy for AVF malformation, outcome: 1.2 Intervention for AVF.

| Study or Subgroup | FIR | | Control | |
| --- | --- | --- | --- | --- |
|  | Events | Total | Events | Total |
| Lin 2007 | 7 | 72 | 16 | 73 |
| Lin 2013 | 4 | 60 | 7 | 62 |

Fig 13. Forest plot of comparison: 1 Far infra-Red phototherapy for AVF malformation, outcome: 1.3 AVF occlusion within 12mo.

| Study or Subgroup | FIR | | Control | |
| --- | --- | --- | --- | --- |
|  | Events | Total | Events | Total |
| Lin 2013 | 3 | 60 | 11 | 62 |
| Chen 2022 | 1 | 50 | 7 | 51 |
