# Supplementary material for: Effects of far infrared therapy in hemodialysis arterio-venous fistula maturation: A meta-analysis
Source: PLoS One. 2024 Aug 28;19(8):e0307586. doi: 10.1371/journal.pone.0307586 (PMC11356441; doi:10.1371/journal.pone.0307586)
Supplement: Attachment — Submitted filename: Response to Reviewers.docx [file pone.0307586.s003.docx]

Dear Editor:

Thank you for your letter of November 28, 2023 and for the opportunity to revise our manuscript. We thank you and the reviewers for your time and effort in reviewing our manuscript. The feedback has been invaluable in improving the content and presentation of the paper. In accordance with your request, the whole manuscript has now been professionally edited.

We have revised our manuscript according to all of the reviewers’ comments. The changes are highlighted in the attached manuscript, and our point-by-point responses are given below. The total word count remains 5471, with 4 tables and 13 figures, but please kindly note we have revised Table 1 to show only essential data and moved the full table to a supplementary file to save space.

------------------------------------------------------------------------------------------------------------------------

**Responses to Reviewer 1:**

**1.Comment:** To the authors: I commend you on the quality of your writing. Although I have some reservations about a four-article review, you have done an excellent job with its composition. I believe those interested in infrared technology for AVFs will find this article to be a pleasant read. Best regards,

**Response:** We thank the reviewer for giving recognition to this article. Indeed, four-article review had its limitation, such as insufficient sample size and statistically power. We did our best to lower the bias and increase the power of evidence by using random model, standardized data assessment with standardized mean difference, and subgroup analysis. The above information is all included in the section on limitations. We thank the reviewer again for his affirmation of this article.

**Responses to Reviewer 2:**

**1.Comment:** I read your manuscript with great interest. Overall idea is good and paper is well established. However I have some major concerns as follows: Meta-analysis should be conducted when a group of studies is sufficiently homogeneous in terms of subjects involved, interventions, and outcomes to provide a meaningful summary. However, it is often appropriate to take a broader perspective in a meta-analysis than in a single clinical trial. The reliability of the conclusion is influenced by factors like the presence of bias, heterogeneity among the studies (PICO), and the overall certainty of the evidence. If there is minimal bias and high certainty in the evidence, the results from these two studies should have the potential to provide a meaningful conclusion. The power analysis should be made.

**Response:** We thank the reviewer for pointing out that power of evidence should be carefully evaluated. Throughout all the results, there were three parameters with high heterogeneity, including assessment flow Qa1, Qa2, and Qa4. We use the power analysis through R language (version 4.3.2 for Windows, 79 megabytes, 64 bit) for evaluate the power of evidence. The result of power analysis was listed in the revised manuscript.

**Responses to Reviewer 3:**

**1.Comment:** I congratulate the authors for their efforts. They conducted a successful systematic review and meta-analysis evaluating the effects of FIR treatment on fistula maturity and patency, including four RCTs. I think that the design, methodology and ethical requirements of the study were appropriate and well written. Although the shortcomings of two similar meta-analyses were mentioned in the introduction, I think that this study does not show very different and strong results from the previous two meta-analyses. Please discuss any real strengths and differences from the other two meta-analyses by highlighting them in the discussion section.

In addition: - On page 10 line 94, in the sentence (2. Participant Type: ESRD patients diagnosed with CKD and AVF,...), AVF is not a diagnosis but a vascular access route for HD in CKD patients. Please edit this sentence.

**Response:** We thank the reviewer for pointing out that the strength and difference from the other two meta-analysis were not clear yet. Nevertheless, although those key parameters presented similar outcome, we have mentioned new data in our article, such as access flow at 1,3 and 6 months (Qa1, Qa2, Qa3), which related to the clinical maturation of arterio-venous fistula. Besides, those key parameters were separated in the two meta-analyses (Bashar et al. mentioned AVF patency and surgical intervention for AVF malfunction; Wan et al. mentioned assessment flow < six months or > six months, AVF diameter, AVF patency, AVF occlusion, needling pain), it was hard to figure out whether all of the parameters were statistically significant or not. In our article, we integrated all the parameters mentioned before, and provided our answer in the article.

And for the part of addition, we thank the reviewer for pointing out these errors. The reviewer is correct, and we apologize for the inappropriate presentation. We have now deleted the misunderstanding words and change the sentence to " 2. Participant Type: patients diagnosed with CKD or ESRD, and receiving regular HD treatment with AVF "

All authors have read and approved the changes made to the manuscript. We hope that the revised paper is now suitable for inclusion in the PLOS one and we look forward to hearing from you.

Yours sincerely,

Pin-Jui Huang, Dr.

Divisions of Urology, Department of Surgery, Ditmanson Medical Foundation Chia-Yi Christian Hospital, Chia-Yi, Taiwan

Tel: +886 952 989113

Email:07639@cych.org.tw
