# Supplementary material for: Effects of far infrared therapy in hemodialysis arterio-venous fistula maturation: A meta-analysis
Source: PLoS One. 2024 Aug 28;19(8):e0307586. doi: 10.1371/journal.pone.0307586 (PMC11356441; doi:10.1371/journal.pone.0307586)
Supplement: Attachment — Submitted filename: Response to Reviewers.docx [file pone.0307586.s004.docx]

Dear Editor:

Thank you for your letter of May 16, 2024 and for the opportunity to revise our manuscript. We thank you and the reviewers for your time and effort in reviewing our manuscript. The feedback has been invaluable in improving the content and presentation of the paper. In accordance with your request, the whole manuscript has now been professionally edited.

We have revised our manuscript according to all of the reviewers’ comments. The changes are highlighted in the attached manuscript, and our point-by-point responses are given below. The total word count remains 5447, with 4 tables and 13 figures.

------------------------------------------------------------------------------------------------------------------------

**Responses to Reviewer 2:**

**1.Comment:** Dear Authors, I reviewed the revised version of the manuscript. I congratulate to you for successful revision. The paper can be published as is.

**Response:** We thank the reviewer for giving recognition to this article. Indeed, four-article review had its limitation, such as insufficient sample size and statistically power. We did our best to lower the bias and increase the power of evidence by using random model, standardized data assessment with standardized mean difference, and subgroup analysis. The above information is all included in the section on limitations. We thank the reviewer again for his affirmation of this article.

**Responses to Reviewer 3:**

**1.Comment:** I would like to thank the authors for their responses to previous suggestions and for their efforts to improve the manuscript accordingly.

**Response:** We thank the reviewer for pointing out the shortcomings of this meta-analysis and the inappropriate presentation previously. We thank the reviewer again for his suggestion to this article.

All authors have read and approved the changes made to the manuscript. We hope that the revised paper is now suitable for inclusion in the PLOS one and we look forward to hearing from you.

Yours sincerely,

Pin-Jui Huang, Dr.

Divisions of Urology, Department of Surgery, Ditmanson Medical Foundation Chia-Yi Christian Hospital, Chia-Yi, Taiwan

Tel: +886 952 989113

Email:07639@cych.org.tw
